# Potential benefits of using a toolkit developed to aid in the adaptation of HTA reports: a case study considering positron emission tomography (PET) and Hodgkin's disease

**DOI:** 10.1186/1478-4505-8-16

**Published:** 2010-05-26

**Authors:** Sheila Turner, Neil Adams, Andrew Cook, Alison Price, Ruairidh Milne

**Affiliations:** 1 NIHR Evaluation, Trials and Studies Coordinating Centre (NETSCC), Alpha House, University of Southampton Science Park, Southampton, SO16 7NS, UK; 2 Public Health Commissioning Team, NHS Southampton City, Trust Headquarters, Oakley Road, Southampton, SO16 4GX, UK

## Abstract

**Background:**

The preparation of HTA reports requires a great deal of time, effort and resource, and there is a desire to improve efficiency, avoid duplication of effort and facilitate the transfer of knowledge between countries. This is of particular importance for countries with more limited resources which have less capacity to produce their own reports. The aim of this study was to investigate the extent of duplication of published Health Technology Assessment (HTA) reports, on the same technology, for the same indication; using positron emission tomography (PET) for lung cancer and Hodgkin's disease as a case study. This was done in order to assess the potential usefulness of a toolkit developed to aid in the adaptation of HTA reports from one context or country to another.

**Methods:**

A systematic search of the National Institute for Health Research (NIHR) CRD HTA database was conducted in June 2008 in order to identify full HTA reports containing information on the use of PET for lung cancer and Hodgkin's disease, written in English, and readily available on the web. The contents of the reports identified were then examined to assess the extent of duplication of content between reports and potential for the use of the toolkit.

**Results:**

From 132 records of HTA reports about PET, 8 reports were identified as fulfilling all the criteria set, and therefore demonstrating potential duplication of effort. All these reports covered four similar domains, technology use, safety, effectiveness and economic evaluation. Five of the reports also considered organisational aspects.

**Conclusions:**

There was some duplication of effort in the preparation of HTA reports concerned with the use of PET for lung cancer and Hodgkin's disease. This is an example of where resource could have been conserved and time saved by the use of a toolkit developed to aid in the adaptation of HTA reports from one context to another.

## Background

The preparation of HTA reports requires a great deal of time, effort and resource. There is a desire to improve efficiency, avoid duplication of effort [1-3] and facilitate the sharing of information between countries. This conservation of resource could be maximised by a process of adaptation, utilising the relevant parts of other HTA reports prepared for use in other countries or contexts [4]. The stage of development and amount of resource available to HTA institutions within member states of the European Union varies considerably; and varies even more worldwide. This process of adaptation could be of particular importance for less well resourced agencies e.g. outside the EU, and when several agencies require information on the same technology at a similar stage of technological development.

The European Network for Health Technology Assessment (EUnetHTA) [5] was established to strengthen links between HTA agencies in Europe and to develop tools to aid in HTA development [6,7]. This project consisted of eight separately managed work packages (WP1-8). Resources such as the International Network of Agencies for Health Technology Assessment (INAHTA) checklist [8] and the Equity-Oriented Toolkit for Health Technology [9] are available to guide those using and preparing HTA reports, however information contained in reports can rarely be used in different contexts without being extracted, updated and adapted [4]. Previous publications have described the development of a toolkit [4,10] and accompanying glossary [11] which were developed to aid the process of adaptation of HTA reports[10,12], as part of the output from work package 5 of the EUnetHTA project 2006-8 [13].

This toolkit is composed of a series of checklists and resources which identify or clarify the relevance, reliability and transferability of data and information from existing reports. It comprises two sections: (i) speedy sifting; a screening tool which would enable 'speedy sifting' of existing HTA reports to assess the relevance of the HTA report for adaptation, and (ii) main toolkit; a more comprehensive tool with questions on reliability and issues regarding transferability. The toolkit can be used to adapt a whole HTA report or parts of it.

The toolkit provides checklists and resources for five HTA report areas of interest which we have termed as domains: technology use and development, safety, efficacy and effectiveness, cost-effectiveness and organisational aspects. The extent to which adaptation is possible depends on the generalisability of the topic under consideration and the different contexts for which the information is required [14-16]. Some domains within these reports are more readily adapted to other contexts. Effectiveness, safety, and technology use are examples of domains which may readily lend themselves to the process; specific issues relating to cultural, legal or political aspects may not be so appropriate for adaptation [4,10].

The toolkit has been subjected to a process of quality assurance testing [4], (HTA partner organisations have tested the toolkit using it to aid in the adaptation of selected reports to meet the needs of their own health services) and has been found useful.

Positron Emission Tomography (PET) is a 3D imaging technique of wide application, which measures physiological function by looking at blood flow, metabolism, neurotransmitters, and radio-labelled drugs [17]. PET is a complex technology; it can operate in several modalities e.g. full ring PET, partial ring PET, and gamma camera PET.

A number of agencies have produced HTA reports on use of the technique for a variety of indications. PET was chosen as a suitable topic for this case study because of this large number of reports and because of the earlier debate concerning reliability of HTA reports concerning PET [18,19]. One clinical use of PET is in the field of oncology to distinguish between benign and malignant tumours. To date some of the strongest evidence of effectiveness and cost effectiveness of the techniques is emerging in relation to lung cancer and Hodgkin's lymphoma [20].

This case study aims to investigate the extent of duplication in HTA reports concerning PET for lung cancer and/or Hodgkin's disease; and to demonstrate the potential usefulness of a tool for the adaptation of HTA reports, with a view to conserving resource. In cases where duplication has occurred, such a toolkit would have been of great benefit had it been available at the time, facilitating sharing of information, and saving time.

## Methods

### Identification of reports

In June 2008 the National Institute for Health Research (NIHR) CRD HTA database [21] was searched for HTA reports about the use of PET. The search terms used were PET or "positron emission tomograph*". There was no limitation placed on language. Reports published more than 10 years ago were excluded because of the stage of development of the technology.

**Figure 1:**
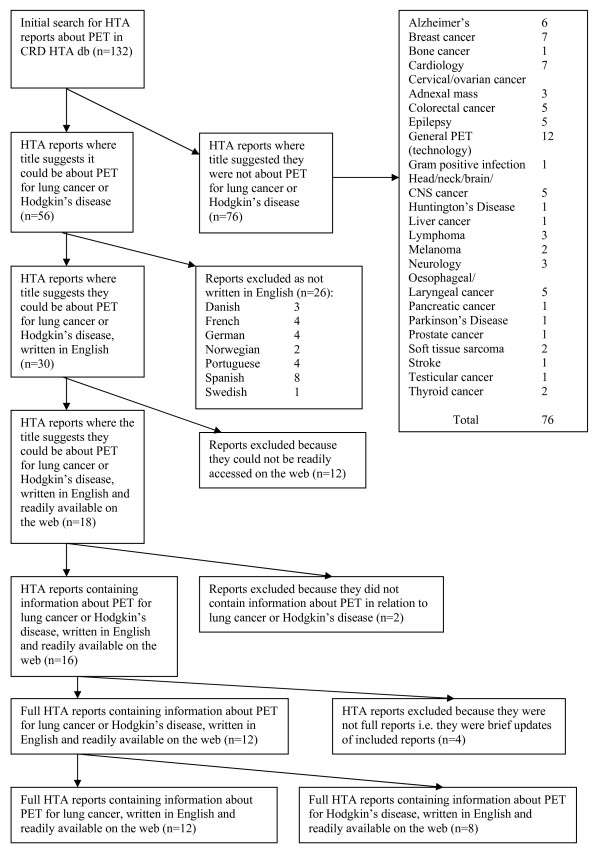
**Identification of full HTA reports written in English, readily available on the internet, containing information regarding the use of PET, in the diagnosis of lung cancer and/or Hodgkin's disease**.

Titles were scanned to identify those reports which might contain information about the use of PET for lung cancer and/or Hodgkin's disease. Reports which clearly related to other areas were listed separately (see Fig 1).

Records were then scanned to establish whether the reports were written in English, or if versions in English were available. (English was specified as a criterion as there was no resource available to translate reports available only in other languages). The CRD database included information regarding language of publication for some reports, for those where this was not clear from the database, attempts were made to locate the reports on the internet and establish in which language they were written. Where reports were written in languages other than English, these were recorded (see Fig 1).

For the reports where the title suggested they could be about PET for lung cancer or Hodgkin's disease, written in English; attempts were made to access full versions of the reports on the internet. If the reports were not readily available over the internet or if they were not full reports but updates of earlier included reports this was also recorded (see Fig 1).

Finally full reports which were written in English and freely available over the internet were examined to establish if they did contain information in relation to the use of PET in the diagnosis of either lung cancer and/or Hodgkin's disease (Fig 1).

This study aimed to devise a pragmatic way of assessing duplication and mimicking what happens in usual practice. Hence pragmatic decisions were made concerning the methods for selection of reports (selecting full reports written in English, available over the internet).

### Examination of reports

The eight reports identified as fulfilling all criteria and containing information on Hodgkin's disease were then examined for information regarding:

i) Date of publication and whether they had cited previous reports identified.

ii) Domains included in the reports. This information is related to the domains included in the adaptation toolkit, and was analysed as an indication of the potential usefulness of the toolkit in these examples.

iii) Purpose of the reports.

## Results

### Identification of reports

132 records of HTA reports about PET were found in the initial search of the CRD HTA database. Reading of the titles enabled 56 records to be selected as potentially containing information about PET in relation to diagnosis of lung cancer or Hodgkin's disease.

Of these 56, 30 were written in or available in English and of those, 18 were readily available on the web. Of those 18, two reports were found not to contain information about lung cancer or Hodgkin's disease. Of the remaining 16 reports four were excluded because they were not full reports but updates reports already included.

This left 12 full reports, written in English, readily available on the web, which contained information concerning the use of PET for the diagnosis of either lung cancer and/or Hodgkin's disease (see Fig 1). Of these 12 all contained information on lung cancer and 8 also contained information on Hodgkin's disease; these 8 reports were designated A-H in chronological order of the date of publication.

### Examination of reports

The 8 reports were published between 1999 and 2007 (see Additional file 1, table S1). The second report published in 1999 cited the first. All the reports published in 2001 cited the two 1999 reports, but not each other. A sixth report was published in 2002, citing only 3 of the earlier 5 reports. The 2005 report cited all from 2001 and one from 1999. The last report only cited one from 2001, and the two reports from 2002 and 2005.

All these reports covered four similar domains, technology use, safety, effectiveness and economic evaluation (see table 1). Five of the reports also considered organisational aspects. These five domains are the domains considered in the adaptation toolkit, and are the sections of the reports which could therefore lend themselves to the process of adaptation, facilitated by the toolkit.

**Table 1:** Date of publication and domains included in reports

Report	Year	Month	Origin	Domains				
				**Technology use & developments**	**Safety**	**Effectiveness (Including efficacy)**	**Economic evaluation**	**Organisational aspects**

A	1999	July	UK	yes	yes	yes	yes	no

B	1999	November	INAHTA members	yes	yes	yes	yes	no

C	2001	Unknown	Québec	yes	yes	yes	yes	yes

D	2001	May	Ontario	yes	yes	yes	yes	no

E	2001	August	Australia	yes	yes	yes	yes	yes

F	2002	Unknown	Scotland	yes	yes	yes	yes	yes

G	2005	October	Belgium	yes	yes	yes	yes	yes

H	2007	November	UK	yes	yes	yes	yes	yes

The purposes for which the reports were written are described below:

#### A [22] 1999. Robert and Milne

This was a three month project which had two objectives: (i) to review the state of knowledge regarding clinical applications of PET, and (ii) to determine the key HTA research questions relating to the use of PET in the UK.

#### B [23] 1999. Adams et al

INAHTA conducted this joint collaboration in response to an increasing global interest in the clinical potential of positron emission tomography (PET). The project documents PET use and related public health coverage in countries represented by INAHTA members and synthesizes technology assessments of PET conducted by INAHTA members and three private US organizations. It considers all PET systems, that is, conventional full ring models, newer partial ring models and SPECT cameras modified for imaging positron emitters.

#### C [24] 2001. Dussault et al

This assessment report was undertaken following a joint request from the Fédération des médecins spécialistes du Québec (FMSQ) and the Conseil québécois de lutte contre le cancer (CQLC) concerning the clinical efficacy of positron emission tomography (PET). The objectives of AÉTMIS's assessment were: (a) to gather hard data on the clinical use of PET in different fields, in particular, oncology, neurology and cardiology; and (b) to make recommendations concerning the possible deployment of PET in Québec.

#### D [25] May 2001. Laupacis

This health technology assessment of Positron Emission Tomography (PET) was requested by the Committee on Technical Fees, a committee consisting of membership from the Ontario Ministry of Health and Long-Term Care (MOHL-TC), the Ontario Medical Association (OMA) and the Ontario Hospital Association (OHA). The Institute for Clinical Evaluative Sciences (ICES) was asked to

(a) review the existing literature about the diagnostic accuracy, effect upon patient outcomes and cost-effectiveness of PET,

(b) identify clinical indications for which PET is likely to be shown to be diagnostically accurate and cost-effective in the near future,

(c) estimate the number of patients in Ontario who may benefit from PET, given current information about its diagnostic accuracy, effectiveness and cost-effectiveness, and

(d) identify areas of clinical research related to PET that are of importance to Ontarians. This report was to consider the clinical use of PET only, not basic research using PET.

#### E [26] August 2001. MSAC

The clinical effectiveness of FDG PET had been assessed previously for 11 indications, in two reviews. This report summarises the assessment of evidence for FDG PET for an additional three specific indications: (a) assessment of patients with lymphoma (Hodgkin's and non-Hodgkin's lymphoma) for staging of disease prior to therapy; (b) assessment of patients with squamous cell carcinoma (SCC) of the head and neck for staging prior to initial definitive treatment; and (c) assessment of patients with sarcoma (soft tissue and bone) for staging and grading of disease

#### F [27] 2002. Bradbury et al

This Health Technology Assessment (HTA) set out with two principal objectives: (a) To determine the role of PET imaging in cancer management: evaluating the clinical and cost effectiveness in terms of impact on patient mortality and morbidity. (b) If PET is found to be clinically and cost effective, to consider the best configuration of PET facilities (and cyclotrons) to serve the Scottish population.

#### G [28] 2005. Cleemput et al.

The main objectives of this HTA report were to: (a) To review the existing evidence on the diagnostic accuracy, clinical effectiveness and cost effectiveness of PET, (b) to describe the current situation of PET in Belgium, including regulation, frequency of use and costs for the national insurance RIZIV/INAMI, (c) to formulate practical recommendations for the organisation of PET services in Belgium based on the existing evidence and data.

#### H [29] 2007. Facey et al.

The aim of this review was to assess the clinical effectiveness of FDG-PET in breast, colorectal, head and neck, lung, lymphoma (Hodgkin's and non-Hodgkin's), melanoma, oesophageal and thyroid cancers. For each cancer, use of FDG-PET to aid management decisions relating to diagnosis, staging/restaging, recurrence, treatment response and radiotherapy (RT) planning were evaluated.

All the reports considered the use of PET and developments, safety, effectiveness and economic evaluation, (see table 1) although Facey et al 2007 (H) [29] state that the "study was not intended to evaluate economic reviews of PET and so did not undertake a search of all economic sources. However, during the systematic review for effectiveness, some studies of cost effectiveness were identified". These studies were subsequently verified against those identified in G [28]. Five of the eight reports considered organisational aspects.

## Discussion

As so many of the reports cited previous reports, the authors must have been aware of the previous work. Clearly there has been at least some duplication of effort, in the preparation of these reports. It was stated in D [25] that while the report was in the process of being written, the authors became aware that L'Agence d'Évaluation des Technolgies et des Modes d'Intervention en Santé (AÉTMIS) in Quebec was also preparing a report on PET scanning [24]. Both groups wrote their reports independently, but shared drafts of their reports, and met once for a face-to-face meeting.

Previous work has described the development of a toolkit to aid the adaptation of HTA reports [4,10]; from one setting, for use in another setting: the objectives being to conserve resource and save time. Some "field testing" of the toolkit has been undertaken in Europe which has been found useful, its' testing and development is currently continuing. The toolkit aims to help the user decide if new work is required or if existing work can be adapted for their purposes by prompting questions concerning the quality and relevance of existing reports. This could be particularly useful for countries with limited resources as it could enable them to make use of information presented by other countries, adapting relevant sections for their own context. The toolkit provides checklists and resources for five HTA report domains: technology use and development, safety, efficacy and effectiveness, cost-effectiveness and organisational aspects. All 8 reports considered here, reviewed the first 4 of the 5 domains; 5 reports also considered organisational aspects.

The four domains considered by all 8 reports contain the type of information most readily adapted between contexts [10], (information in the fifth domain, organisational aspects, is more difficult to adapt to specific countries or contexts). This case study provides a good example of where a toolkit to aid adaptation would have been useful had it been available. For example the teams preparing the two Canadian reports in 2001 (reports C and D), could have used the toolkit to adapt information for their own purposes, sharing resource and reducing the amount of effort required to produce two new reports; report F published in 2002 cites one of the previous reports from 2001 (report D), yet did not adapt information from the earlier report. It is anticipated that use of the toolkit in the future will save time, effort and resource.

Avoiding duplication of HTA reports has been advocated as a means of avoiding duplicating effort unnecessarily; however, it should be acknowledged that sometimes there is a need to produce another HTA report on the same or a very similar topic. This may appear to be duplication, but the need is justified. Reasons for this may include i) older HTA reports on a relatively new technology may be very broad in their nature, a new report may be needed to cover certain aspects in more detail; ii) rapid evolution of the technology requiring updated evaluation; iii) better methods of assessing diagnostic tests becoming available; iv) re-analysis of data using improved statistical methods or v) instructions from commissioning institution(s) which preclude HTA teams from building on previous work e.g. NICE.

One strength of this study is the in depth systematic analysis of the available data. The duplication of HTA reports is mentioned frequently in the literature; however, we have not been able to identify any other studies which have systematically analysed the data to test the extent to which this is the case for an individual technology.

Three possible weaknesses of this study are considered here. Firstly only reports available in English were included. Reports written in other languages may have been relevant, but we lacked resource to be able to translate them, and so the information contained in them was unfortunately not available to us. Also, only those reports readily available on the web were considered, paper copies could have been requested, however, hard copy is not always readily available.

Searches were restricted to the CRD database; however, this is the most comprehensive source of completed and ongoing HTA research from around the world. It is produced in collaboration with the International Network of Agencies for Health Technology Assessment (INAHTA) Secretariat, and contains summaries of ongoing and completed projects conducted by the INAHTA member agencies as well as records reporting completed technology assessments carried out by other HTA organisations. Importantly, this database is free to search and so would be available to less well resourced agencies.

This study only considered one technology, PET, which is a complex, rapidly evolving diagnostic technology employed in many disease areas. The results may well have been different if a less complex technology relevant to perhaps a single disease area had been considered. However, use of the toolkit is still relevant, as it prompts the user to consider and make judgements about issues such as technology use and development, and as such the toolkit would still be useful as an aid in considering information for possible adaptation. This could be an area for future research.

## Supplementary Material

Additional file 1 **Table S1**. HTA reports containing information on the use of PET in the diagnosis of Hodgkin's disease, cited in subsequent reports Click here for file

## Conclusions

There was some duplication of effort in the preparation of HTA reports concerned with the use of PET for lung cancer and Hodgkin's disease. This is an example of where resource could have been conserved and time saved by the use of a toolkit developed to aid in the adaptation of HTA reports from one context or country to another.

## List of abbreviations

EUnetHTA: European Network for Health Technology Assessment; HTA: Health Technology Assessment; PET: positron emission tomography.
