# Supplementary material for: Potential benefits of using a toolkit developed to aid in the adaptation of HTA reports: a case study considering positron emission tomography (PET) and Hodgkin's disease
Source: Health Res Policy Syst. 2010 May 26;8:16. doi: 10.1186/1478-4505-8-16 (PMC2887859; doi:10.1186/1478-4505-8-16)
Supplement: Additional file 1 — Table S1. HTA reports containing information on the use of PET in the diagnosis of Hodgkin's disease, cited in subsequent reports [file 1478-4505-8-16-S1.DOC]

## Table S1 - HTA reports containing information on the use of PET in the diagnosis of Hodgkin’s disease, cited in subsequent reports

|  |  | Previous reports | | | | | | | |
| --- | --- | --- | --- | --- | --- | --- | --- | --- | --- |
|  |  | A  (July 99) | B  (Nov 99) | C  (2001) | D  (May 01) | E  (Aug 01) | F  (2002) | G  (Oct 05) | H  (Nov 07) |
| Report under consideration | A (July 1999) |  |  |  |  |  |  |  |  |
|  | B (Nov1999) | **yes** |  |  |  |  |  |  |  |
|  | C (2001) | **yes** | **yes** |  | no | no |  |  |  |
|  | D (May 2001) | **yes** | **yes** | no |  |  |  |  |  |
|  | E (Aug 2001) | **yes** | **yes** | no | no |  |  |  |  |
|  | F (2002) | **yes** | **yes** | no | **yes** | no |  |  |  |
|  | G ( Oct 2005) | no | **yes** | **yes** | **yes** | **yes** | no |  |  |
|  | H (Nov 2007) | no | no | no | **yes** | no | **yes** | **yes** |  |
